# Predicting Complete Response to Neoadjuvant Chemotherapy in Muscle-Invasive Bladder Cancer

**DOI:** 10.3390/cancers15010168

**Published:** 2022-12-28

**Authors:** Hiroko Miyagi, Elizabeth Kwenda, Brian H. Ramnaraign, Jonathan A. Chatzkel, Wayne G. Brisbane, Padraic O’Malley, Paul L. Crispen

**Affiliations:** 1 Department of Urology, University of Florida, Gainesville, FL 32611, USA; 2 Department of Medicine, University of Florida, Gainesville, FL 32611, USA

**Keywords:** urothelial carcinoma, radical cystectomy, neoadjuvant chemotherapy, complete response

## Abstract

**Simple Summary:**

Bladder cancer is the second most common urologic malignancy. Current standard of care for muscle-invasive bladder cancer is cisplatin-based neoadjuvant chemotherapy followed by radical cystectomy. There is an unmet need to predict which patients will benefit from neoadjuvant chemotherapy as treatment results in toxicities associated with therapy as well as delays to radical cystectomy. This review summarizes several predictors of complete response to neoadjuvant chemotherapy as well as current clinical trials to aid urologists and oncologists treating bladder cancer.

**Abstract:**

Muscle-invasive bladder cancer is a life-threatening disease best managed with multimodal therapy. Neoadjuvant chemotherapy prior to cystectomy significantly improves survival with the greatest benefit noted in patients with a complete pathologic response noted at cystectomy. While radical cystectomy is currently an important part of the treatment plan, surgical morbidity remains high. Accurate prediction of complete responses to chemotherapy would enable avoiding the morbidity of radical cystectomy. Multiple clinical, pathologic, molecular, and radiographic predictors have been evaluated. Clinical and standard pathologic findings have not been found to be accurate predictors of complete response. To date, tumor genomic findings have been the most promising and have led to multiple clinical trials to evaluate if bladder preservation is possible in select patients. Radiomics has shown initial promise with larger validation series needed. These predictors can be further characterized as treatment specific and non-treatment specific. With the potential changing landscape of neoadjuvant therapy prior to radical cystectomy and the limitations of individual predictors of a complete response, a panel of several biomarkers may enhance patient selection for bladder preservation. The aim of this review is to summarize predictors of complete response to neoadjuvant chemotherapy.

## 1. Introduction

Bladder cancer is the second most common urologic cancer. Approximately 81,000 new cases of bladder cancer (BC) are expected in 2022. Approximately 70% of patients present with NMIBC at time of diagnosis and 30% of patients have MIBC at presentation. Risk factors for BC include age, sex, smoking, exposure to aromatic amines, aristolochic acid, diet (red meat intake, nitrites used in processed meat) and catabolites excreted in the urine [1,2]. Optimum treatment of muscle-invasive bladder cancer (MIBC) requires multidisciplinary care with radical cystectomy (RC) being considered a critical component. Neoadjuvant cisplatin-based chemotherapy prior to radical cystectomy is recommended for appropriately selected patients. The largest survival benefit associated with neoadjuvant chemotherapy (NAC) is seen in patients who achieve a complete response (CR), pT0, with no residual tumor at time of radical cystectomy. However, radical cystectomy is a morbid procedure associated with several complications and impact on quality of life. Given the morbidity associated with radical cystectomy and the ability to achieve a CR with NAC, the ability to predict those patients who may achieve CR with NAC may allow for bladder preservation in appropriately selected patients. To date, there are no established predictors of response to NAC. In this article, we summarize the recommended NAC regimens for MIBC, rate of complete response to NAC, and potential predictors of complete response to NAC.

## 2. Neoadjuvant Chemotherapy in Urothelial Cell Bladder Cancer

Randomized clinical trials have demonstrated a survival advantage for patients with localized MIBC who receive NAC prior to RC compared with RC alone [3]. Platinum-based NAC has shown a 5% improvement in overall survival at five years, improving survival from 45% to 50% [4]. This was equivalent to a 13% relative reduction in risk of death [4]. The survival benefits seen with NAC are likely a consequence of treatment of micro metastatic disease [5]. NAC does not adversely affect the chances of proceeding with RC nor increase the complications related to surgery compared to those who proceed with upfront RC [3]. Additionally, no differences in 30-day complication rates were seen in those who underwent NAC prior to RC than those who underwent RC alone [6]. There are limited data on the survival of patients who refuse or are unfit to undergo RC following NAC. A series by Robins et al. evaluated 41 patients who received NAC but did not undergo immediate cystectomy [7]. The majority of patients had clinical T2 disease prior to NAC and had a negative cystoscopy, cytology and no evidence of disease on CT scan following NAC. Five-year disease-free survival was 58% and cystectomy-free rate was 79%. Of the patients with recurrent disease, most recurred within one year of NAC (89%) and had stage I disease (84%). These results suggest that carefully selected patients may be adequately treated with NAC alone if they are willing to accept the potential need for future intravesical therapy and salvage cystectomy.

## 3. Pathologic Complete Response and Overall Survival

SWOG 8710 was a pivotal phase III clinical trial demonstrating improved rates of CR with neoadjuvant MVAC (methotrexate, vinblastine, doxorubicin, cisplatin) compared to RC alone. In the MVAC cohort, 38% of RC specimens achieved CR, compared to 15% in the RC only cohort (*p* < 0.001) [3]. Importantly, CR noted in the RC specimen was associated with improvement in overall survival, with a median survival of 77 months in the MVAC cohort compared to only 46 months in RC only cohort (*p* = 0.05) [3]. When stratified by tumor stage at presentation, a larger survival benefit was noted in those with T2 disease compared to T3-T4a disease with a median survival of 105 months versus 75 months for T2 disease and 65 months versus 24 months for T3-T4a disease (*p* = 0.05) [3]. Similar to SWOG 8710, Hermens et al. found a significant benefit to partial response rates (<pT1) with use of NAC. Partial response rates of cT2 patients who received NAC compared to RC only were 43% versus 25% (*p* < 0.001). Patients with cT3-4a disease also had significantly higher partial response rate with NAC compared to RC only, 37% versus 8.2% (*p* < 0.001) [8].

Rosenblatt et al. noted similar 5-year overall survival rates of 88.2% associated with CR to NAC compared to that noted in SWOG 8710, 5-year overall survival rate of 85% [3,9]. CR was significantly lower in those who did not undergo NAC with 5-year overall survival rates of only 57.1% [9]. Petrelli et al. found that CR is associated with 55% lower risk of death and an 81% lower risk of recurrence compared to those with residual disease [10]. Taken together, the results of these studies demonstrate that CR to NAC is associated with a significant survival advantage in patients with MIBC.

## 4. Potential Predictors of Complete Response to Neoadjuvant Chemotherapy

The established CR rates with NAC, and preliminary data demonstrating acceptable survival in patients with clinical CR refusing cystectomy, have led to the evaluation of multiple CR predictors. Box 1 presents potential predictors which will be described in greater detail.

Box 1 **Potential Predictors of Complete Response to Neoadjuvant Chemotherapy.**

Clinical presentation

Neoadjuvant chemotherapy regimen

Repeat transurethral resection

Molecular subtype of urothelial carcinoma

Tumor genomics

Gene expression models

Circulating tumor DNA

Radiomics

## 5. Clinical Staging

Complete transurethral resection of all visible bladder tumors has been associated with improved outcomes in patients undergoing trimodal therapy. Conversely, there is no known benefit in CR when grossly resecting all visible tumors prior to NAC. In a series of 100 patients undergoing cisplatin-based NAC and radical cystectomy, CR was noted in 29% of patients with grossly complete resections and 20% in patients with incomplete resections prior to NAC [11]. Of note, the small sample size of this study may not allow adequate evaluation of the difference in CR dependence on complete transurethral resection. Additionally, CR can be noted with complete TURBT alone with a T0 rate of 15% in patients undergoing RC alone in SWOG 8710 [3,12].

Hydronephrosis has been associated with adverse pathologic stage and outcomes following cystectomy. A retrospective study by Pokuri et al. of 30 patients undergoing NAC with hydronephrosis at initial presentation found that the absence of hydronephrosis favored CR; however, this did not reach statistical significance (34% versus 13%, *p* = 0.15) [12]. Additionally, no difference in CR rate was noted in patients with clinical T2 compared to T3/4 disease, 29% versus 26%, prior to NAC. The impact of clinical stage on response to NAC was also evaluated by Hermans et al. who noted pathologic downstaging (≤T1) rates of 43% in cT2 disease compared to 37% in more advanced disease [8]. These limited data suggest that NAC should be recommended regardless of hydronephrosis and/or clinical stage.

Additional pretreatment radiographic features of bladder tumors have been explored. Nguyen et al. published a prospective study evaluating the role of multi-parametric MRI in evaluating tumor heterogeneity as a predictor for chemosensitivity [11]. Increased microcellular heterogeneity seen on ADC maps was associated with increased likelihood of tumor resistance to NAC [13]. These findings suggest that MRI may play an additional role in predicting bladder cancer response to NAC.

## 6. Primary Versus Secondary Muscle-Invasive Bladder Cancer

The classification of primary and secondary bladder cancer is based upon patients having a history of stage I bladder cancer prior to developing MIBC. Primary MIBC is assigned when patients have MIBC at the time of initial diagnosis, while secondary MIBC is assigned when patients progress from ≤T1 disease. Pietzak et al. retrospectively analyzed differences in pathologic response rates to NAC in those with primary versus secondary MIBC. Pietzak et al. found that those with secondary MIBC have significantly lower pathologic response (≤T1) rates to NAC (26% versus 45%, *p* = 0.02) [14]. Furthermore, secondary MIBC was associated with decreased recurrence-free, cancer-specific and overall survival in this series. With these findings, the authors hypothesized that the lower CSS may be due to a delay in definitive treatment, increasing the risk of micro metastatic disease in those undergoing NAC.

## 7. Impact of Neoadjuvant Chemotherapy Regimen on Complete Response

Outcomes data on NAC prior to RC are derived from a mixed population of patients who received mainly MVAC or gemcitabine and cisplatin (GC). Both regimens are considered adequate options for advanced bladder cancer; however, GC was shown to have improved toxicity profile with similar efficacy profiles leading to increased usage of GC over MVAC [15]. To evaluate if selection of NAC regimen has an impact on CR, Yin et al. performed a metanalysis that supports the use of cisplatin-based NAC for MIBC patients [5]. In total, 3285 patients were included with a significant overall benefit associated with NAC (HR: 0.82, *p* < 0.001) and an absolute survival benefit of 8% at 5 years. Additionally, rates of CR to GC or MVAC were comparable: 25.7% versus 25.6% [5]. Zargar et al. performed a multicenter retrospective analysis on 935 patients undergoing NAC prior to RC and found no difference in CR rates between patients receiving GC and MVAC, 23.9% versus 24.5% (*p* = 0.2) [15]. In contrast to these series, the retrospective series by Payton et al. noted a significant increase in the CR rate of ddMVAC (41%) compared with GC (25%) [16]. This suggests that the dosing schedule for NAC may have a significant impact on CR rates. However, compelling evidence that supports the equivalence of CR rates between ddMVAC and GC is noted in SWOG 1314 [17]. This trial randomized 237 patients between NAC with ddMVAC and GC. The comparison of CR rates between the two regimens was a secondary endpoint in this trial demonstrating no significant difference with CR rates for ddMVAC at 28% versus GC at 30%. These results were supported by the VESPER trial which also failed to note a significant difference in CR rates between patients treated with ddMVAC (42%) and GC (36%) [18]. It is of note that although CR rates did not show a significant difference between the two cisplatin-based therapies, the 3-year progression-free survival favors ddMVAC compared to GC (66% vs. 56%, *p* = 0.025) [19]. With the apparent equipoise in CR noted between regimens, selection of NAC should be focused on appropriate patient selection and preference of the treating medical oncologist.

## 8. Assessment of Complete Response following Neoadjuvant Chemotherapy

Pathologic assessment of CR has not been standardized since most patients undergo RC following NAC. The use of routine transurethral resection of bladder tumor (TURBT) alone may not be sufficient based on the results of SWOG 0219 [20]. In this single arm trial, all patients received neoadjuvant gemcitabine, paclitaxel and carboplatin. TURBT was performed on all patients upon completion of NAC. Patients without clinical CR noted on TURBT underwent immediate cystectomy. Patients with clinical CR on TURBT underwent observation or immediate cystectomy. In the group of patients noted to have a clinical CR on TURBT and went to cystectomy, 60% were noted to have residual disease and all had T2 or greater disease. While the NAC regimen in this trial is no longer considered appropriate, the study does raise concerns on the use of TURBT alone to determine CR following NAC. More contemporary series have also noted poor accuracy of post-NAC TURBT pathology in predicting cystectomy pathology. Becker et al. noted that 32% of patients were incorrectly down-staged on post-NAC TURBT. This was associated with a negative predictive value of 62% and sensitivity of 27% [21]. In another series of 157 patients evaluating repeat staging by TURBT, by Kukreja et al. noted persistent disease in 64% of cystectomy specimens [22]. In patients who received NAC, muscle-invasive disease was noted in 51% and node-positive disease was noted in 1.3% despite being cT0 on precystectomy TURBT.

## 9. Molecular Subtypes of Muscle-Invasive Bladder Cancer

Additional predictors of CR need to be explored with the noted limitations of clinical staging, NAC regimen, and repeat TURBT in predicting CR to NAC. Detection of molecular and genomic markers to predict chemosensitivity would reduce the toxicity and potential delay in care in patients unlikely to benefit from NAC. Another benefit of molecular subtyping may be to guide selection of optimal therapy. Molecular subtypes of MIBC may have a role in predicting sensitivity and resistance to chemotherapy as well as to targeted therapy. Several molecular classification systems have been proposed to define molecular subtypes of MIBC [23]. Within each classification system, molecular subtypes are associated with unique clinicopathologic and genomic features. Additionally, these classification systems have been found to be consistent across platforms and between institutions. Routine use of such molecular subtype classification systems could improve treatment selection. Of the subtypes, basal MIBCs are enriched with squamous and sarcomatoid features and often associated with advanced stage and metastatic disease at presentation [24]. Although basal bladder cancers are aggressive, they are believed to be highly sensitive to cisplatin-based chemotherapy [24]. Molecular characterization of pre-NAC TUR specimens by Seiler et al. found that patients with basal tumors derive the most benefit from NAC (3yr OS: 77.8% in NAC cohort vs. 49.2% non-NAC cohort, *p* < 0.001) indicating the importance of NAC in this cohort [25]. Luminal MIBC subtype may be categorized into luminal and p53-like. Luminal subtype tends to have more papillary features and is rich in FGFR3. Luminal bladder cancers appear to respond to NAC while p53-like tumors are believed to be resistant to chemotherapy [26]. Contrarily, Seiler et al.’s findings of luminal tumors having the best OS amongst the molecular subtypes regardless of receipt of NAC may negate the need for NAC in this subtype [25]. Woonyoung et al. studied gene expression profiles of responders and non-responders to NAC and found chemosensitive basal tumors had high infiltration of immune cells (B and T lymphocytes) [24]. Unfortunately, no biomarkers to predict responders were found in the two subtypes of luminal bladder cancers in this analysis. Sjodahl et al. evaluated response to NAC based on molecular subtype in 149 patients [27]. Genomically unstable tumors demonstrated increased CR rates (52%) compared to urothelial-like (31%) and basal/squamous (21%) subtypes [27]. Currently, molecular subtype has not been consistently associated with CR to NAC and there is not enough experience with the subtypes to incorporate routine use as predictors of response [23].

A recent review on metabolomic analysis highlights the relevance of evaluating differences in metabolites found in biologic samples due to alterations in catabolic processes in cancer cells. Metabolomics is defined as the study of metabolites in a sample (urine, blood, tissue) to identify potential biomarkers. Promising studies identifying urinary metabolites have increased the sensitivity of BC detection over standard urine cytology [28]. Additionally, metabolomics is leading to advances in improving detection of bladder cancer recurrence, predicting treatment outcomes and cancer-specific survival [28].

## 10. Tumor Genomics and Gene Expression Profiles

Chemosensitivity based on specific gene mutations noted in tumor sample has been evaluated by several investigators. Groenendijk et al. compared gene mutations from pre-NAC tumor samples obtained from patients with a CR to NAC to patients with ≥pT2 disease noted at cystectomy [29]. Next-generation sequencing of 178 genes was completed noting an increased number of ERBB2 mutations in patients with CR (9/38) compared to non-responders (0/33). Additionally, an increased number of ERCC2 missense mutations were noted in the CR cohort, but was not statistically significant. While statistically insignificant in this series, DNA damage repair (DDR) gene alterations have been associated with improved response to cisplatin-based NAC in several other series. Van Allen et al. noted a significant association of response to cisplatin-based NAC in patients with somatic ERCC2 mutations [30]. This finding was validated in a series of 55 patients noting pathologic down staging in 40% of patients with somatic ERCC2 mutations compared to 7% of patients without an ERCC2 mutation [31]. A prospective phase II study by Iyer et al. evaluated rate of pathologic downstaging in patients treated with neoadjuvant dose dense GC [32]. Forty-nine patients were enrolled, with pathologic downstaging (<pT2) noted in 57% of patients and CR (T0) in 15% of patients. DDR gene alterations were evaluated in 32 patients with adequate tissue samples. DDR gene alterations were noted in 28% of patients and were associated with a positive predictive value for pathologic downstaging (89%) and improved 2-year recurrence-free survival. The second series by Miron et al. evaluated 58 patients treated with neoadjuvant ddMVAC or GC for association between DDR gene mutations and clinical outcomes [33]. The three DDR genes evaluated in this series were ATM, RB1, and FANCC. A mutation of at least one of these three genes was noted in 64% of responders to NAC compared to only 15% in non-responders.

Several ongoing prospective trials are evaluating if DDR gene alterations can identify patients who can safely avoid cystectomy following NAC. In Alliance A031701 (NCT03609216), all patients with T2-4aN0M0 urothelial carcinoma receive neoadjuvant dose dense GC. Patients with DDR alterations undergo TURBT after completing NAC; if <cT1 disease is noted, patients undergo surveillance; if >cT1 disease is noted, patients can undergo cystectomy or chemoradiation therapy. Patients without DDR alterations do not undergo TURBT after NAC and are treated with radical cystectomy or chemoradiation. The second trial RETAIN (NCT02710734) registers patients with T2-T4aN0M0 disease. All patients receive neoadjuvant MVAC followed by a restaging TURBT. Future treatment is based upon DDR gene mutation status and TURBT pathology. Patients with DDR gene mutations and that are cT0 undergo active surveillance. DDR mutation-positive patients with residual stage I disease and DDR mutation-negative cT0 patients can choose to undergo either intravesical therapy, chemoradiation or cystectomy. Patients with cT2 disease can choose radical cystectomy or chemoradiation. Patients with >cT3 disease will undergo radical cystectomy. The design of both these trials allows patients to avoid radical cystectomy based on DDR gene mutation status and restaging TURBT after cisplatin-based NAC. If these trials are positive, the results may greatly expand treatment options for MIBC.

Tumor gene expression profiles have also been evaluated as a means of predicting response to NAC. One such profile that has been evaluated is coexpression extrapolation, COXEN [17]. COXEN was developed based upon gene expression and drug sensitivity of 60 well established cell lines. Comparison of gene expression profiles between cell lines and tumor samples is used to generate a COXEN score to predict treatment response. SWOG 1314 was designed to evaluate the association of COXEN score for the NAC regimens MVAC and GC with pathologic response at cystectomy. Neither MVAC nor GC COXEN scores were predictive of CR rate at cystectomy. However, a pooled analysis applying the GC COXEN score to both treatment arms was associated with pathologic down staging (pT0 and ≤pT1 disease). Despite the negative results of this trial, it did provide valuable data comparing pathologic response rates between NAC regimens and may serve as a platform to evaluate future biomarkers.

In addition to the genomic markers described above, multiple other molecular based biomarkers have been evaluated. An excellent review by Tse et al. outlines the promise and challenges of molecular biomarkers for the prediction of CR with NAC [26]. Since the publication of this prior review, several other molecular biomarkers have been evaluated and are shown in Table 1. Continued evaluation of such biomarkers with validation across other series and inclusion in clinical trials evaluating NAC should be supported.

## 11. Circulating Tumor DNA

Circulating tumor DNA (ctDNA) has demonstrated promise to guide treatment decisions in several solid organ tumors. The potential role of ctDNA in predicting CR in patients with MIBC has been evaluated by Christensen et al. [41]. In their series, ctDNA levels were evaluated prior to NAC, after NAC, and after radical cystectomy in 68 patients. Only 43% of patients were noted to be ctDNA positive prior to NAC. Patients who remained ctDNA positive prior to and after cystectomy had significantly worse recurrence-free and overall survival compared to patients who were ctDNA negative. While the small number of patients who were ctDNA negative prior to NAC limits the evaluation of predicting response to NAC, the findings may suggest that patients who remain ctDNA positive after NAC may not benefit from radical cystectomy. Another potential promising method for assessing tumor DNA levels is in the evaluation of urine tumor DNA, utDNA. Chauhan et al. noted a significant association of utDNA levels following NAC with CR [42]. Detectable utDNA levels after NAC were highly correlated with the presence of residual disease with a sensitivity of 81% and specificity of 81%. Future development and validation of these initial series evaluating circulating tumor DNA are warranted.

## 12. Radiomics

The field of radiomics is rapidly expanding and has potential in evaluating response to NAC in patients with MIBC. Table 2 lists several series evaluating radiomics in predicting CR to NAC. Common among these series is a small sample size and need for validation. However, there appears great potential for such technology, especially if it is used in combination with a genomic or molecular biomarker as described above.

## 13. Future Directions

Results from current clinical trials evaluating bladder preservation following NAC may have a tremendous impact on patient care. This is primarily based on the prediction of CR and avoiding radical cystectomy in select patients. However, treating clinicians and physicians must understand that T0 disease noted at cystectomy is not always associated with cure. In fact, node-positive disease is noted in up to 7.5% of pT0 patients and disease recurrence is noted in as many of 10% despite pT0N0 pathology at cystectomy [48]. Furthermore, most promising predictors of CR to NAC currently under evaluation are specific to cisplatin-based treatment regimens. Nevertheless, many patients are ineligible for cisplatin-based NAC and many non-cisplatin-based NAC regimens are currently being evaluated. Initial results from trials evaluating the use of checkpoint inhibitors in the neoadjuvant setting are encouraging, reporting CR rates of up to 45%. Multiple predictors of CR in patients receiving neoadjuvant pembrolizumab have been evaluated, including immune signatures, interferons, tumor mutational burden, combined positive score, and MRI-based radiomics [49,50,51,52]. This potential change in the neoadjuvant landscape may lead to treatment selection based upon predictive biomarkers, increase the importance of predictors that are not treatment specific, and assessment of multiple predictors when evaluating treatment response.

## Figures and Tables

**Table 1:** Molecular Based Biomarkers of Complete Response to Neoadjuvant Chemotherapy.

Author	Biomarker Evaluated	Prediction of CR
O’Donnell, P.H. et al. [34]	Nine germline single nucleotide polymorphisms	Polymorphisms in RARS and GALNTL4 significant in discovery cohort, but not significant in validation cohort
Font, A. et al. [35]	KRT5/6, KRT14, GATA3, and FOXA1	Increased CR in patients with BASQ-like tumors
Nassif, E.F. et al. [36]	Immunoscore	High Immunoscore associated with increased CR
Jutte, H. et al. [37]	KRT20, KRT5, ESR1, ERBB2 mRNA expression	Elevated expression of KRT20, ERBB2 and ESR1 associate with CR
Koskinen, I. et al. [38]	Clinical features, Ki-67, p53, HER-2, and EGFR	No significant association with CR noted
Ornstein, M.C. et al. [39]	Peripheral circulating myeloid derived suppressor cells	Decreased levels of peripheral circulating myeloid derived suppressor cells associated with CR
Bazargani, S.T. et al. [40]	Serum CA-125, CA 19-9, and CEA	Increased downstaging noted in patients with normalized serum markers after NAC

**Table 2:** Radiographic Predictors of Complete Response to Neoadjuvant Chemotherapy.

Author	Imaging Modality	Model	Prediction of CR
Choi, S.J. et al. [43]	CT	Imaging based model including tumor shape, clinical stage and tumor size	Training cohort: AUC 0.85 (95% CI, 0.78–0.93) Validation cohort: AUC 0.75 (95% CI, 0.60–0.86)
Choi, S.J. et al. [44]	CT	Bladder wall thickening and enhancement characteristics noted on urothelial phase	Reader 1: AUC 0.85 (95% CI, 0.76–0.93) Reader 2: AUC 0.85 (95% CI)
Ahmed, S.A. et al. [45]	MRI	Dynamic and diffusion weighted MRI	Reader 1: AUC 0.981 Reader 2: AUC 0.971
Soubra, A. et al. [46]	FDG-PET/CT	Change in maximum SUV on FDG-PET/CT	Sensitivity for CR 75% Negative Predictive Value for CR 89%
Cha, K.H. et al. [47]	CT	Decision support system utilizing deep-learning neural networks and radiomics	AUC improved with support system compared to clinician alone

## References

[1] Aveta A., Cacciapuoti C., Barone B., Di Zazzo E., Del Giudice F., Maggi M., Ferro M., Terracciano D., Busetto G.M., Lucarelli G. (2022). The Impact of Meat Intake on Bladder Cancer Incidence: Is It Really a Relevant Risk?. Cancers.

[2] Siegel R.L., Miller K.D., Jemal A. (2018). Cancer statistics, 2018. CA Cancer J. Clin..

[3] Grossman H.B., Natale R.B., Tangen C.M., Speights V.O., Vogelzang N.J., Trump D.L., White R.W.d., Sarosdy M.F., Wood D.P., Raghavan D. (2003). Neoadjuvant Chemotherapy plus Cystectomy Compared with Cystectomy Alone for Locally Advanced Bladder Cancer. N. Engl. J. Med..

[4] Vale C. (2003). Neoadjuvant chemotherapy in invasive bladder cancer: A systematic review and meta-analysis. Lancet.

[5] Yin M., Joshi M., Meijer R.P., Glantz M., Holder S., Harvey H.A., Kaag M., Fransen van de Putte E.E., Horenblas S., Drabick J.J. (2016). Neoadjuvant Chemotherapy for Muscle-Invasive Bladder Cancer: A Systematic Review and Two-Step Meta-Analysis. Oncologist.

[6] Milenkovic U., Akand M., Moris L., Demaegd L., Muilwijk T., Bekhuis Y., Laenen A., van Cleynenbreugel B., Everaerts W., van Poppel H. (2019). Impact of neoadjuvant chemotherapy on short-term complications and survival following radical cystectomy. World J. Urol..

[7] Robins D., Matulay J., Lipsky M., Meyer A., Ghandour R., DeCastro G., Anderson C., Drake C., Benson M., McKiernan J.M. (2018). Outcomes Following Clinical Complete Response to Neoadjuvant Chemotherapy for Muscle-invasive Urothelial Carcinoma of the Bladder in Patients Refusing Radical Cystectomy. Urology.

[8] Hermans T.J.N., Voskuilen C.S., Deelen M., Mertens L.S., Horenblas S., Meijer R.P., Boormans J.L., Aben K.K., van der Heijden M.S., Pos F.J. (2019). Superior efficacy of neoadjuvant chemotherapy and radical cystectomy in cT3-4aN0M0 compared to cT2N0M0 bladder cancer. Int. J. Cancer.

[9] Rosenblatt R., Sherif A., Rintala E., Wahlqvist R., Ullén A., Nilsson S., Malmström P.U. (2012). Pathologic downstaging is a surrogate marker for efficacy and increased survival following neoadjuvant chemotherapy and radical cystectomy for muscle-invasive urothelial bladder cancer. Eur. Urol..

[10] Petrelli F., Coinu A., Cabiddu M., Ghilardi M., Vavassori I., Barni S. (2014). Correlation of pathologic complete response with survival after neoadjuvant chemotherapy in bladder cancer treated with cystectomy: A meta-analysis. Eur. Urol..

[11] Ghandour R.A., Kusin S., Wong D., Meng X., Singla N., Freifeld Y., Bagrodia A., Margulis V., Sagalowsky A., Lotan Y. (2020). Does grossly complete transurethral resection improve response to neoadjuvant chemotherapy?. Urol. Oncol. Semin. Orig. Investig..

[12] Pokuri V.K., Syed J.R., Yang Z., Field E.P., Cyriac S., Pili R., Levine E.G., Azabdaftari G., Trump D.L., Guru K. (2016). Predictors of Complete Pathologic Response (pT0) to Neoadjuvant Chemotherapy in Muscle-invasive Bladder Carcinoma. Clin. Genitourin. Cancer.

[13] Nguyen H.T., Mortazavi A., Pohar K.S., Zynger D.L., Wei L., Shah Z.K., Jiaa G., Knopp M.v. (2017). Quantitative Assessment of Heterogeneity in Bladder Tumor MRI Diffusivity: Can Response be Predicted Prior to Neoadjuvant Chemotherapy?. Bladder Cancer.

[14] Pietzak E.J., Zabor E.C., Bagrodia A., Armenia J., Hu W., Zehir A., Funt S., Audenet F., Barron D., Maamouri N. (2019). Genomic Differences Between “Primary” and “Secondary” Muscle-invasive Bladder Cancer as a Basis for Disparate Outcomes to Cisplatin-based Neoadjuvant Chemotherapy. Eur. Urol..

[15] Zargar H., Espiritu P.N., Fairey A.S., Mertens L.S., Dinney C.P., Mir M.C., Krabbe L.M., Cookson M.S., Jacobsen N.E., Gandhi N.M. (2015). Multicenter assessment of neoadjuvant chemotherapy for muscle-invasive bladder cancer. Eur. Urol..

[16] Peyton C.C., Tang D., Reich R.R., Azizi M., Chipollini J., Pow-Sang J.M., Manley B., Spiess P.E., Poch M.A., Sexton W.J. (2018). Downstaging and Survival Outcomes Associated with Neoadjuvant Chemotherapy Regimens among Patients Treated with Cystectomy for Muscle-Invasive Bladder Cancer. JAMA Oncol..

[17] Flaig T.W., Tangen C.M., Daneshmand S., Alva A., Lerner S.P., Scott Lucia M., McConkey D.J., Theodorescu D., Goldkorn A., Milowsky M.I. (2021). A randomized phase II study of coexpression extrapolation (COXEN) with neoadjuvant chemotherapy for bladder cancer (SWOG S1314; NCT02177695). Clin. Cancer Res..

[18] Pfister C., Gravis G., Fléchon A., Soulié M., Guy L., Laguerre B., Mottet N., Joly F., Allory Y., Harter V. (2021). Randomized Phase III Trial of Dose-dense Methotrexate, Vinblastine, Doxorubicin, and Cisplatin, or Gemcitabine and Cisplatin as Perioperative Chemotherapy for Patients with Muscle-invasive Bladder Cancer. Analysis of the GETUG/AFU V05 VESPER Trial Secondary Endpoints: Chemotherapy Toxicity and Pathological Responses[Formula presented]. Eur. Urol..

[19] Pfister C., Gravis G., Fléchon A., Chevreau C., Mahammedi H., Laguerre B., Guillot A., Joly F., Soulié M., Allory Y. (2022). Dose-Dense Methotrexate, Vinblastine, Doxorubicin, and Cisplatin or Gemcitabine and Cisplatin as Perioperative Chemotherapy for Patients With Nonmetastatic Muscle-Invasive Bladder Cancer: Results of the GETUG-AFU V05 VESPER Trial. J. Clin. Oncol..

[20] deVere White R.W., Lara P.N., Goldman B., Tangen C.M., Smith D.C., Wood D.P., Hussain M.H.A., Crawford E.D. (2009). A Sequential Treatment Approach to Myoinvasive Urothelial Cancer: A Phase II Southwest Oncology Group Trial (S0219). J. Urol..

[21] Becker R.E.N., Meyer A.R., Brant A., Reese A.C., Biles M.J., Harris K.T., Netto G., Matoso A., Hoffman-Censits J., Hahn N.M. (2021). Clinical Restaging and Tumor Sequencing are Inaccurate Indicators of Response to Neoadjuvant Chemotherapy for Muscle-invasive Bladder Cancer [Formula presented]. Eur. Urol..

[22] Kukreja J.B., Porten S., Golla V., Ho P.L., Noguera-Gonzalez G., Navai N., Kamat A.M., Dinney C.P.N., Shah J.B. (2018). Absence of Tumor on Repeat Transurethral Resection of Bladder Tumor Does Not Predict Final Pathologic T0 Stage in Bladder Cancer Treated with Radical Cystectomy. Eur. Urol. Focus.

[23] Satyal U., Sikder R.K., Mcconkey D., Plimack E.R., Abbosh P.H. (2019). Clinical implications of molecular subtyping in bladder cancer. Curr. Opin. Urol..

[24] Choi W., Czerniak B., Ochoa A., Su X., Siefker-Radtke A., Dinney C., McConkey D.J. (2014). Intrinsic basal and luminal subtypes of muscle-invasive bladder cancer. Nat. Rev. Urol..

[25] Seiler R., Ashab H.A.D., Erho N., van Rhijn B.W.G., Winters B., Douglas J., van Kessel K.E., Fransen van de Putte E.E., Sommerlad M., Wang N.Q. (2017). Impact of Molecular Subtypes in Muscle-invasive Bladder Cancer on Predicting Response and Survival after Neoadjuvant Chemotherapy [Figure presented]. Eur. Urol..

[26] Tse J., Ghandour R., Singla N., Lotan Y. (2019). Molecular predictors of complete response following neoadjuvant chemotherapy in urothelial carcinoma of the bladder and upper tracts. Int. J. Mol. Sci..

[27] Sjödahl G., Abrahamsson J., Holmsten K., Bernardo C., Chebil G., Eriksson P., Johansson I., Kollberg P., Lindh C., Lövgren K. (2022). Different Responses to Neoadjuvant Chemotherapy in Urothelial Carcinoma Molecular Subtypes. Eur. Urol..

[28] di Meo N.A., Loizzo D., Pandolfo S.D., Autorino R., Ferro M., Porta C., Stella A., Bizzoca C., Vincenti L., Crocetto F. (2022). Metabolomic Approaches for Detection and Identification of Biomarkers and Altered Pathways in Bladder Cancer. Int. J. Mol. Sci..

[29] Groenendijk F.H., de Jong J., Fransen Van De Putte E.E., Michaut M., Schlicker A., Peters D., Velds A., Nieuwland M., van den Heuvel M.M., Kerkhoven R.M. (2016). ERBB2 Mutations Characterize a Subgroup of Muscle-invasive Bladder Cancers with Excellent Response to Neoadjuvant Chemotherapy. Eur. Urol..

[30] van Allen E.M., Mouw K.W., Kim P., Iyer G., Wagle N., Al-Ahmadie H., Zhu C., Ostrovnaya I., Kryukov G.V., O’connor K.W. (2014). Somatic ERCC2 mutations correlate with cisplatin sensitivity in muscle-invasive urothelial carcinoma. Cancer Discov..

[31] Liu D., Plimack E.R., Hoffman-Censits J., Garraway L.A., Bellmunt J., Allen E van Rosenberg J.E. (2016). Clinical validation of chemotherapy response biomarker ERCC2 in muscle-invasive urothelial bladder carcinoma. JAMA Oncol..

[32] Iyer G., Balar A.V., Milowsky M.I., Bochner B.H., Dalbagni G., Machele Donat S., Herr H.W., Huang W.C., Taneja S.S., Woods M. (2018). Multicenter prospective phase ii trial of neoadjuvant dose-dense gemcitabine plus cisplatin in patients with muscle-invasive bladder cancer. J. Clin. Oncol..

[33] Miron B., Hoffman-Censits J.H., Anari F., O’Neill J., Geynisman D.M., Zibelman M.R., Kutikov A., Viterbo R., Greenberg R.E., Chen D. (2020). Defects in DNA Repair Genes Confer Improved Long-term Survival after Cisplatin-based Neoadjuvant Chemotherapy for Muscle-invasive Bladder Cancer. Eur. Urol. Oncol..

[34] O’Donnell P.H., Alanee S., Stratton K.L., Garcia-Grossman I.R., Cao H., Ostrovnaya I., Plimack E.R., Manschreck C., Ganshert C., Smith N.D. (2016). Clinical Evaluation of Cisplatin Sensitivity of Germline Polymorphisms in Neoadjuvant Chemotherapy for Urothelial Cancer. Clin. Genitourin. Cancer.

[35] Font A., Domènech M., Benítez R., Rava M., Marqués M., Ramírez J.L., Pineda S., Domínguez-Rodríguez S., Gago J.L., Badal J. (2020). Immunohistochemistry-based taxonomical classification of bladder cancer predicts response to neoadjuvant chemotherapy. Cancers.

[36] Nassif E.F., Mlecnik B., Thibault C., Auvray M., Bruni D., Colau A., Compérat E., Bindea G., Catteau A., Fugon A. (2021). The immunoscore in localized urothelial carcinoma treated with neoadjuvant chemotherapy: Clinical significance for pathologic responses and overall survival. Cancers.

[37] Jütte H., Reike M., Wirtz R.M., Kriegmair M., Erben P., Tully K., Weyerer V., Eckstein M., Hartmann A., Eidt S. (2021). Krt20, krt5, esr1 and erbb2 expression can predict pathologic outcome in patients undergoing neoadjuvant chemotherapy and radical cystectomy for muscle-invasive bladder cancer. J. Pers. Med..

[38] Koskinen I., Boström P.J., Taimen P., Salminen A., Tervahartiala M., Sairanen J., Erickson A., Mirtti T. (2021). Prediction of neo-adjuvant chemotherapy response in bladder cancer: The impact of clinical parameters and routine biomarkers. Scand. J. Urol..

[39] Ornstein M.C., Diaz-Montero C.M., Rayman P., Elson P., Haywood S., Finke J.H., Kim J.S., Pavicic P.G., Lamenza M., Devonshire S. (2018). Myeloid-derived suppressors cells (MDSC) correlate with clinicopathologic factors and pathologic complete response (pCR) in patients with urothelial carcinoma (UC) undergoing cystectomy. Urol. Oncol. Semin. Orig. Investig..

[40] Bazargani S.T., Clifford T.G., Djaladat H., Schuckman A.K., Wayne K., Miranda G., Cai J., Sadeghi S., Dorff T., Quinn D.I. (2019). Association between precystectomy epithelial tumor marker response to neoadjuvant chemotherapy and oncological outcomes in urothelial bladder cancer. Urol. Oncol. Semin. Orig. Investig..

[41] Christensen E., Birkenkamp-Demtröder K., Sethi H., Shchegrova S., Salari R., Nordentoft I., Wu H.T., Knudsen M., Lamy P., Lindskrog S.V. (2019). Early detection of metastatic relapse and monitoring of therapeutic efficacy by ultra-deep sequencing of plasma cell-free DNA in patients with urothelial bladder carcinoma. J. Clin. Oncol..

[42] Chauhan P.S., Chen K., Babbra R.K., Feng W., Pejovic N., Nallicheri A., Harris P.K., Dienstbach K., Atkocius A., Maguire L. (2021). Urine tumor DNA detection of minimal residual disease in muscle-invasive bladder cancer treated with curative-intent radical cystectomy: A cohort study. PLoS Med..

[43] Choi S.J., Park K.J., Heo C., Park B.W., Kim M., Kim J.K. (2021). Radiomics-based model for predicting pathological complete response to neoadjuvant chemotherapy in muscle-invasive bladder cancer. Clin. Radiol..

[44] Choi S.J., Park K.J., Lee G., Kim M hyun Kim J.K. (2020). Urothelial phase CT for assessment of pathologic complete response after neoadjuvant chemotherapy in muscle-invasive bladder cancer. Eur. J. Radiol..

[45] Ahmed S.A., Taher M.G.A., Ali W.A., Ebrahem M.A.E.S. (2021). Diagnostic performance of contrast-enhanced dynamic and diffusion-weighted MR imaging in the assessment of tumor response to neoadjuvant therapy in muscle-invasive bladder cancer. Abdom. Radiol..

[46] Soubra A., Gencturk M., Froelich J., Balaji P., Gupta S., Jha G., Konety B.R. (2018). FDG-PET/CT for Assessing the Response to Neoadjuvant Chemotherapy in Bladder Cancer Patients. Clin. Genitourin. Cancer.

[47] Cha K.H., Hadjiiski L.M., Cohan R.H., Chan H.P., Caoili E.M., Davenport M.S., Samala R.K., Weizer A.Z., Alva A., Kirova-Nedyalkova G. (2019). Diagnostic Accuracy of CT for Prediction of Bladder Cancer Treatment Response with and without Computerized Decision Support. Acad. Radiol..

[48] Tilki D., Svatek R.S., Novara G., Seitz M., Godoy G., Karakiewicz P.I., Kassouf W., Fradet Y., Fritsche H.M., Sonpavde G. (2010). Stage pT0 at radical cystectomy confers improved survival: An international study of 4,430 patients. J. Urol..

[49] Necchi A., Bandini M., Calareso G., Raggi D., Pederzoli F., Farè E., Colecchia M., Marandino L., Bianchi M., Gallina A. (2020). Multiparametric Magnetic Resonance Imaging as a Noninvasive Assessment of Tumor Response to Neoadjuvant Pembrolizumab in Muscle-invasive Bladder Cancer: Preliminary Findings from the PURE-01 Study. Eur. Urol..

[50] Bandini M., Gibb E.A., Gallina A., Raggi D., Marandino L., Bianchi M., Ross J.S., Colecchia M., Gandaglia G., Fossati N. (2020). Does the administration of preoperative pembrolizumab lead to sustained remission post-cystectomy? First survival outcomes from the PURE-01 study☆. Ann. Oncol..

[51] Bandini M., Ross J.S., Raggi D., Gallina A., Colecchia M., Lucianó R., Giannatempo P., Farè E., Pederzoli F., Bianchi M. (2021). Predicting the pathologic complete response after neoadjuvant pembrolizumab in muscle-invasive bladder cancer. J. Natl. Cancer Inst..

[52] Necchi A., Raggi D., Gallina A., Madison R., Colecchia M., Lucianò R., Montironi R., Giannatempo P., Farè E., Pederzoli F. (2020). Updated Results of PURE-01 with Preliminary Activity of Neoadjuvant Pembrolizumab in Patients with Muscle-invasive Bladder Carcinoma with Variant Histologies. Eur. Urol..

